# The association between cannabis use and brain reward anticipation: a 12-month longitudinal study of adults and adolescents who use cannabis and age-matched controls

**DOI:** 10.1038/s41386-026-02377-3

**Published:** 2026-02-26

**Authors:** Martine Skumlien, Simiao Wang, Tom P. Freeman, Molly Eddison, Kat Petrilli, Matthew B. Wall, Claire Mokrysz, H. Valerie Curran, Will Lawn

**Affiliations:** 1 Department of Addictions, Institute of Psychiatry, Psychology & Neuroscience, King’s College London, London, UK; 2 Addiction and Mental Health Group (AIM), Department of Psychology, University of Bath, Bath, UK; 3 Department of Neuroimaging, Institute of Psychiatry, Psychology & Neuroscience, King’s College London, London, UK; 4 Perceptive, London, UK; 5 Faculty of Medicine, Imperial College London, London, UK; 6 Clinical Psychopharmacology Unit, Clinical, Educational and Health Psychology Department, University College London, London, UK; 7 Department of Psychology, Institute of Psychiatry, Psychology & Neuroscience, King’s College London, London, UK

**Keywords:** Motivation, Addiction

## Abstract

Substance use has been associated with blunted brain responses to non-drug rewards, but findings in people who use cannabis are mixed. Adolescents may be uniquely vulnerable to cannabis-related disruption to reward processing due to ongoing neuromaturation, but longitudinal research is lacking. In this longitudinal fMRI study, we compared brain measures of reward anticipation in 46 adolescents (16–17 years) and adults (26–29 years) who used cannabis (1–7 days/week) and 50 age-matched controls with the Monetary Incentive Delay task at baseline and 12-month follow-up. Region of interest (ROI) analyses adjusted for cigarette/roll-up use, depression, and risk-taking found that reward anticipation activity decreased in the right (*p* = 0.05, *η*_*p*_^*2*^ = 0.04) and left (*p* = 0.02, *η*_*p*_^*2*^ = 0.05) ventral striatum from baseline to follow-up in participants who used cannabis compared with control participants. These effects remained in unadjusted models and when including only participants who consistently used or abstained from cannabis during the study period. There were no significant interactions between the cannabis user-group and age-group, or between the user-group, age-group, and time. There were also no cannabis user-group main or interaction effects in full sample ROI analyses for the thalamus, insula, or supplementary motor area, or in exploratory whole-brain analyses. The current results suggest that cannabis use may be associated with reductions in non-drug reward anticipation activity in the ventral striatum, a key part of the brain’s reward system. However, there was no evidence of adolescent resilience or vulnerability to cannabis-related changes in brain reward anticipation activity.

## Introduction

Adolescence is a critical neurodevelopmental period, with rapid maturation of fronto-striatal circuits, which comprise key parts of the brain’s reward system [1, 2]. The endocannabinoid system also continues to mature into adulthood and plays an important role in neurodevelopmental processes [3, 4]. The main psychoactive compound in cannabis, Δ^9^-tetrahydrocannabinol (THC), acts on the endocannabinoid system as a partial agonist at the cannabinoid-1 receptor (CB1R) [5, 6]. Therefore, this developmental window may confer heightened vulnerability to potential neurobiological effects of cannabis exposure on various cognitive and psychological outcomes, including reward processing [7–9].

Reward anticipation refers to the psychological process of anticipating upcoming rewards based on previously learned stimulus-reward associations [10]. Perturbed reward processing, underpinned by alterations in the mesocorticolimbic dopamine pathway, is theorised to be a cardinal brain pathology of substance use disorder [11]. For instance, several neuroscientific theories, supported by empirical neuroimaging evidence, propose that brain responses to the anticipation of non-drug rewards are blunted in addiction [12–15]. There is a strong rationale for a similar pattern to occur with cannabis use, especially as CB1Rs are found in high density in regions that play important roles in reward anticipation [16, 17].

The Monetary Incentive Delay (MID) task is the most widely used measure of reward anticipation during fMRI. While the majority of cross-sectional studies using the MID task have not found consistent differences in reward region-specific activity between people who use cannabis (PWUC) and non-using controls [18], a longitudinal investigation by Martz et al. (2016) [19] found that greater cannabis use predicted reduced nucleus accumbens activation during reward anticipation at two- and four-year follow-ups in 108 young adults. More recently, Macedo et al. (2024) [20] observed no associations between cannabis use at 19 and 22 years and reward anticipation activity at 14, 19, and 22 years in a sample of 318 participants, although in this study, cannabis use was defined at a relatively low threshold of ≥6 times in the previous year and ≥1 time in the previous month.

Based on the rationale that the developing adolescent reward neurocircuitry and endocannabinoid system may be particularly vulnerable to cannabis-related harms, we would expect the long-term impact of chronic cannabis consumption on the reward system to be stronger in adolescents than in adults. However, only one study has directly compared reward processing in adults and adolescents who use cannabis [21]. That study, which used cross-sectional baseline data from the same study that we report on here, did not find any differences in reward anticipation activity between adult and adolescent PWUC and age-matched controls. Critically, however, no longitudinal studies using the MID task have investigated the association between cannabis use and reward anticipation in adolescents. Therefore, it remains unclear whether cannabis use is associated with changes in the developing adolescent reward system over time, and whether adolescent use poses an augmented risk of reward system changes relative to adult use.

This study presents the first longitudinal fMRI investigation directly comparing adolescent and adult PWUC with age-matched controls on reward anticipation using the MID task. We pre-registered [22] two hypotheses: (i) PWUC would show reduced activation over time in the right ventral striatum, left ventral striatum, right thalamus, right anterior insula, and right supplementary motor area (SMA) compared with controls, and (ii) this reduction would be more pronounced in adolescents than in adults.

## Methods

### Design

This was a longitudinal fMRI study using data from the CannTeen project. For full details on methods, please see the CannTeen study protocol [23]. The current study compared reward anticipation activity assessed with the MID task at baseline and 12-month follow-up in adult and adolescent PWUC and controls. We therefore have between-subjects factors of User-Group (PWUC and controls), Age-Group (adolescents and adults), and Time (baseline/0 months and follow-up/12 months). We have previously published cross-sectional baseline results on the MID task from the CannTeen study [21].

### Participants

We recruited 140 adult (26–29 years) and adolescent (16–17 years) PWUC and non-using controls (*n* = 35 in each group) from the Greater London area via school assemblies, physical posters and flyers, and social media advertisements. These age ranges were selected because cannabis use typically starts around 16 years and is highest among adolescents and young adults <30 years in the UK [24] and because neuromaturation often is considered to stabilise around the mid-20s [1]. Key inclusion criteria were having used cannabis at least once per week (averaged over the past three months) prior to taking part in the study for the PWUC group and ≤10 lifetime occasions of cannabis use for the control group. To isolate the impact of adolescent cannabis use, the adult PWUC could not have used cannabis frequently before the age of 18. Full inclusion and exclusion criteria are reported in Supplemental Table 1. All participants provided written informed consent. The study was conducted in line with the Declaration of Helsinki and was approved by the University College London (UCL) ethics committee (project ID 5929/003).

### Power analysis

This study used data from the larger CannTeen MRI study, which was powered to detect a minimum effect size of *d* = 0.68 for a cross-sectional difference in hippocampal volume between PWUC and controls [25] at *a* = 0.05 and power = 0.95, necessitating a minimum of 116 participants. With 96 participants (see below), we had 80% power to detect a small-to-medium effect size of *f* = 0.14 for the interaction between Time and User-Group (*a* = 0.05, correlation between repeated measures = 0.5, nonsphericity correction = 1).

### Procedure

Participants were invited to complete five behavioural sessions at the UCL Clinical Psychopharmacology Unit and two MRI sessions at the Invicro (now ‘Perceptive’) MRI research facility, Hammersmith, London. The demographic, drug use, and mental health data included in this study were collected at the behavioural sessions. Participants completed an instant saliva drug test (Alere DDSV 703 or ALLTEST DSD-867MET/C, which tested for cocaine, THC, opiates, amphetamine, methamphetamine, and benzodiazepines) and a Lion Alcometer 500 breathalyser and self-reported abstinence at the start of all sessions, to confirm no recent use of alcohol or cannabis (≥12 hours) or other illicit drugs (≥48 hours).

The first CannTeen study session took place on the 1st of November 2017, and the final one on the 16th of April 2021. The baseline MRI session was typically completed within two weeks, and always within two months of the baseline behavioural session. We originally aimed for participants to complete the follow-up MRI session 12 months after the first. However, this requirement had to be relaxed as we had to pause data collection due to the COVID-19 pandemic lockdown initiated in the UK in March 2020. The mean gap between the first and second MRI scan was 1.07 years (standard deviation, *sd* = 0.16), with a minimum of 0.83 and a maximum of 1.76 years, and >84% completed their second MRI session within 1.25 years.

### Measures

Brain reward anticipation activity was assessed with the MID task during fMRI [26]. The current version of the task included win and neutral trials, but no loss trials. Full details are in the Supplemental Methods. Cannabis, tobacco, alcohol, and other drug use were assessed with the timeline follow-back [27, 28]. Covariates in the current analyses were baseline scores on the Risk-Taking 18 (RT-18) Questionnaire [29], baseline and follow-up scores on the Beck Depression Inventory (BDI) [30], and days per week of cigarette/roll-up use at baseline and follow-up. These were chosen a priori due to their putative associations with both cannabis use and reward processing [31–33]. Three participants had missing values for days per week of cigarette/roll-up use and two participants had missing BDI scores at follow-up (corresponding to the fifth behavioural testing session). To avoid excluding these participants, their scores were imputed using values from the latest of the other four behavioural sessions for which the participant had valid data (session two for two participants, session four for one participant).

### fMRI data acquisition, preprocessing, and first-level analysis

MRI data were collected with a 3.0 T Siemens Verio scanner. T_2_* images were acquired using a multiband gradient echo Echo-Planar Imaging (EPI) sequence [34]. T_1_-weighted structural images were acquired using a Magnetization Prepared Rapid Gradient Echo (MPRAGE) sequence [35]. The acquisition sequences and all other aspects of the set-up (task, response boxes, etc.) were identical for the baseline and follow-up sessions. Preprocessing, first-level analyses, and second-level analyses were performed in FSL [36] using FEAT [37, 38]. Full MRI acquisition parameters and preprocessing steps are in Supplemental Methods.

There were two explanatory variables: anticipation of win outcomes and anticipation of neutral outcomes. These were implemented in a General Linear Model, by convolving their respective onsets with a gamma function model of the hemodynamic response. Motion parameters (standard + temporal derivatives + squared + quadratic) and temporal derivatives were included as regressors-of-no-interest. The FILM pre-whitening procedure was used to account for temporal autocorrelation, and a high-pass filter (100 seconds cut-off) was used to remove low-frequency noise. Reward anticipation was examined with the win anticipation > neutral anticipation contrast, which is the most commonly analysed contrast in studies using the MID task [39].

### Statistical analyses

Analyses were pre-registered to the Open Science Framework [22]. Behavioural and ROI analyses were performed in SPSS 31 and R 4.5.1 [40]. Whole-brain analyses were performed in FSL FEAT.

#### Behavioural analyses

The main behavioural outcome on the MID task was mean reaction times (RTs) for win and neutral trials. These were analysed in a fully factorial analysis of variance (ANOVA) with within-subjects factors Time (baseline, follow-up) and Trial-Type (win, neutral), and between-subjects factors User-Group (PWUC, control) and Age-Group (adolescent, adult). As hit rates (% hit targets) were calibrated to 50%, these were not analysed.

#### Whole-brain analyses

Second-level analyses were performed with FSL FLAME. We first examined mean blood-oxygen-level-dependent responses across all participants in separate whole-brain one-sample *t*-tests for the baseline and follow-up sessions. The main effect of Time was explored using a paired-samples t-test. To test the remaining main, two-way interaction, and three-way interaction effects, we first performed two sets of mid-level fixed effects analyses to compute (i) average baseline and follow-up activation and (ii) the difference between baseline and follow-up activation for each participant. The results of both mid-level models were passed up to separate higher-level two-way between-subjects ANOVAs with factors User-Group, Age-Group, and the User-Group*Age-Group interaction. The first, using the ‘average’ mid-level results, was used to test the main effects of User-Group and Age-Group and their interaction, and the second, using the ‘difference’ mid-level results, was used to test the two-way and three-way interactions with Time. In all models, cluster-level statistics were used, with a cluster-defining threshold of *Z* = 3.1 and a multiple test corrected cluster-extent threshold of *a* = 0.05. Regions were labelled using the Harvard-Oxford cortical and subcortical structural atlases [41–43].

#### Region of interest analyses

The region of interest (ROI) analyses were the main analyses used to test our hypotheses. ROIs were the right ventral striatum, left ventral striatum, right thalamus, right anterior insula, and right SMA. These were the five regions with the highest activation likelihood estimate from a large meta-analysis of MID reward anticipation [39]. ROI masks were defined by constructing 6 mm radii spheres around the coordinates with peak activation (see the Supplemental Methods) and used to extract unstandardised beta-values from the baseline and follow-up scans. The ROI betas were then included as dependent variables in separate unadjusted linear mixed models with a random intercept for participant ID and the within-subjects factor Time, between-subjects factors User-Group and Age-Group, and all two and three-way interactions, and in adjusted models that also included covariates baseline RT-18, BDI (time-varying), and cigarette/roll-up days per week (time-varying).

Cannabis use changed for some participants over the 12 months of the study, with some control participants reporting use at later visits, and some PWUC reducing or stopping their use. Therefore, we also ran sensitivity ROI analyses including only those participants who consistently met the criteria for the PWUC group (using cannabis ≥1 day/week) or control group (using cannabis 0 days/week) at each behavioural testing session that they completed. We additionally performed exploratory bivariate Pearson correlations between ROI change scores (follow-up values minus baseline values) and (i) mean days/week of cannabis use for the completed follow-up behavioural sessions (session 2–5) and (ii) change in days/week of cannabis use between baseline and follow-up (mean days/week for session 2–5 minus days/week at session 1).

Finally, we computed intraclass correlation coefficients (ICC) for all ROIs to determine whether the fMRI MID reward anticipation activity was measured reliably over time (test-retest reliability). ICC estimates were calculated using SPSS 31 based on a single-rating, absolute agreement, two-way mixed-effects model (ICC(3, 1)) as recommended by Koo and Li (2016) [44].

## Results

### Participant characteristics

Of the 140 participants recruited at baseline, 110 completed the follow-up MRI session. Seven participants were excluded due to abnormal or missing behavioural data and seven were excluded due to excessive head movement or other MRI artefacts (see Supplemental Methods), leaving a final sample of 96 participants.

Participant characteristics are displayed in Table 1. We conducted chi-square tests and independent samples *t*-tests to explore whether participants who completed the follow-up session and were included in analyses (*n* = 96) differed from those who were not (*n* = 44). There were no differences between the groups in any of the variables displayed in Table 1 or in ROI reward anticipation activity (all *p*s > 0.05). Adolescent PWUC used cannabis on average 3.04 days per week (*sd* = 1.94) at baseline and 3.15 days per week (*sd* = 2.34, one participant missing) at follow-up. Adult PWUC used cannabis on average 4.04 days per week (*sd* = 2.04) at baseline and 2.92 days per week (*sd* = 2.35, one participant missing) at follow-up.

**Table 1:** Participant characteristics.

	Adolescent PWUC (*n* = 26)	Adult PWUC (*n* = 20)	Adolescent controls (*n* = 24)	Adult controls (*n* = 26)	Group differences
Demographics and covariates					
**Gender**, *n*(%)					ns
Female	12 (46.2%)	9 (45.0%)	12 (50.0%)	13 (50.0%)	
Male	14 (53.8%)	11 (55.0%)	12 (50.0%)	13 (50.0%)	
**Age in years**, mean(sd)	17.08 (0.52), 16.26–17.95	27.51 (1.00), 26.22–29.68	17.15 (0.50), 16.23–17.98	27.34 (1.00), 26.03–29.74	Adults > Adolescents***
**Maternal education**, *n*(%)					ns
Below undergraduate degree	11 (42.3%)	9 (45.0%)	11 (45.8%)	17 (65.4%)	
Undergraduate degree or above	14 (53.8%)	11 (55.0%)	13 (54.2%)	8 (30.8%)	
**BDI**, mean (sd), range					
Baseline	9.62 (6.27), 1–31	6.45 (8.22), 0–31	9.29 (6.18), 0–26	7.38 (8.60), 0–39	ns
Follow-up	10.23 (10.19), 0–38	8.55 (11.20), 0–46	13.67 (14.46), 0-57	6.38 (7.61), 0-30	ns
**RT-18**, mean (sd)	11.58 (3.11), 7–16	7.60 (4.12), 3–15	7.92 (3.99), 0–16	7.08 (4.34), 0–16	PWUC > Controls* Adolescents > Adults**
**Cigarette/roll-up use, days/week**, mean (sd), range					
Baseline	2.57 (2.92), 0–7	1.82 (2.91), 0–7	0.57 (1.71), 0–6.5	0.38 (1.40), 0-7	PWUC > Controls***
Follow-up	2.49 (2.98), 0–	2.08 (3.01), 0–7	0.59 (1.96), 0-7	0.49 (1.75), 0-7	PWUC > Controls***
**Alcohol use, days/week**, mean (sd), range	0.89 (0.79), 0–3.25	1.68 (1.38), 0–5.25	0.57 (0.62), 0-–2.08	1.54 (1.30), 0–5.25	Adults > Adolescents***
**Other illicit drug use, monthly use**, *n*(%)					PWUC > Controls*** Adolescents > Adults*
Yes	15 (57.7%)	2 (10.0%)	0	0	
No	11 (42.3%)	18 (90.0%)	24 (100.0%)	24 (100.0%)	
Cannabis use					
**Ever use**, *n*(%)			20 (83.3%)	26 (100.0%)	Adults > Adolescents*
**Number of lifetime uses**, mean (sd), range			3.37 (3.29), 0–10	5.27 (3.31), 1–10	ns
**Days/week of use**, mean (sd), range	3.04 (1.94), 1–6.92	4.04 (2.05), 0.75–6.92			ns
**Grams used per day of use**, mean (sd), range	0.94 (0.82), 0.15–4.00	0.77 (0.83), 0.03–3.00			ns
**Age of first ever use**, mean (sd)	14.46 (1.11), 12–16	17.36 (3.42), 13–24			Adults > Adolescents**
**Age of first weekly use**, mean (sd)	15.64 (1.12), 13.25–17.67	22.27 (2.93), 17.00–27.67			Adults > Adolescents***
**CUDIT**, mean (sd), range	14.62 (5.95), 5–26	12.40 (5.60), 3–23			ns
**DSM-5 severe CUD**, *n*(%)	11 (42.3%)	5 (25.0%)			ns

* *p* <0.05.

** *p* < 0.01.

*** *p* < 0.001.

*BDI* Beck Depression Inventory, *CUD* Cannabis Use Disorder, *CUDIT* Cannabis Use Disorder Identification Test, *DSM* Diagnostic and Statistical Manual of Mental Disorders, *ns* not significant, *PWUC* people who use cannabis, *sd* standard deviation, *RT-18* Risk-Taking 18.

Group differences were investigated with 2 × 2 analyses of variance, independent samples *t*-tests, or chi-square tests of independence. Age was assessed at the baseline imaging session. All other variables were assessed at the baseline behavioural session plus the fifth follow-up behavioural session for the time-varying covariates BDI and days/week of cigarette/roll-up use.

### MID task

#### Behavioural results

The ANOVA for RT showed a significant effect of Trial-Type (*F*(1, 92) = 87.50, *p* < 0.001, *η*_*p*_^*2*^ = 0.49), Time ((1, 92) = 6.85, *p* = 0.01, *η*_*p*_^*2*^ = 0.07), and Time*Trial-Type*Age-Group (*F*(1, 92) = 8.72, *p* = 0.004, *η*_*p*_^*2*^ = 0.09). *Post hoc* Bonferroni-corrected *t*-tests showed that RTs were faster for win (mean = 237 ms) than neutral (mean = 243 ms) trials across all groups and timepoints (all *p*s < 0.001). There was also a significant decrease in RTs for win trials among adolescents from baseline to follow-up (*p* = 0.002).

#### Whole-brain results

The one-sample *t*-tests showed a large cluster of activation for both baseline and follow-up scans, with peaks in the dorsal striatum, partially overlapping with the ventral striatum and thalamus, the anterior insula, the anterior cingulate cortex, and the cerebellum (Supplementary Fig. 1). This pattern of activation mirrors that found in a large meta-analysis of the MID task [39] and therefore serves to validate our acquisition and analysis procedures.

The paired-samples *t*-test for the main effect of Time showed a significant decrease in activity from baseline to follow-up in three clusters with peaks in the frontal pole and the anterior superior frontal gyrus (Supplementary Table 2, Supplemental Fig. 2). The ANOVA using the mid-level ‘difference’ models showed a significant Time*Age-Group interaction in two clusters with peaks in the right cerebellum and the left superior frontal gyrus (Supplementalry Table 2). For both clusters, the adolescents showed increased activity from baseline to follow-up, whereas the adults showed decreased activity (Supplementary Fig. 3). There were no other significant main or interaction effects.

#### ROI results

**Fig. 1:**
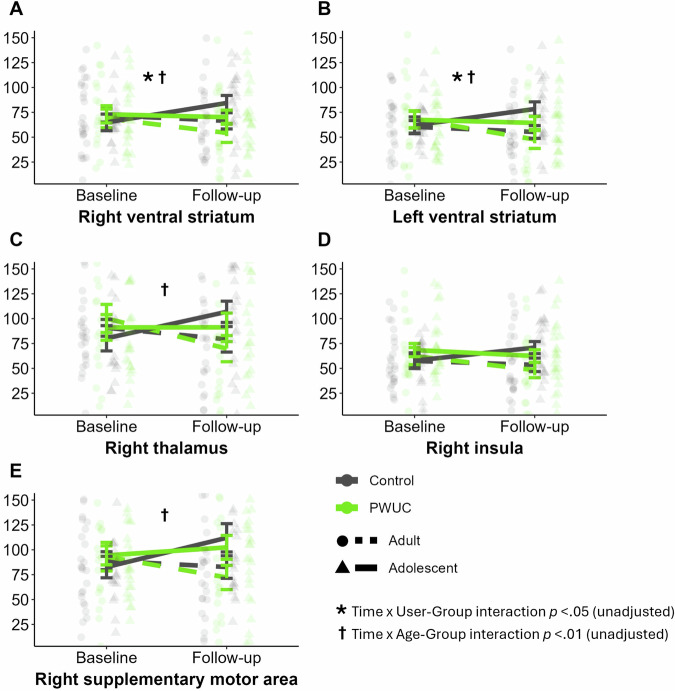
Reward anticipation activity (beta-values) in regions of interest at baseline and follow-up by User-Group and Age-Group. Group differences were explored with linear mixed models. Unadjusted models are presented, but we also repeated the analyses controlling for Beck Depression Inventory scores at baseline and follow-up, days per week of cigarette/roll-up use at baseline and follow-up, and Risk-Taking 18 scores at baseline. **A** There were significant interactions between Time and User-Group (*p* = 0.05, *η*_*p*_^*2*^ = 0.04) and Time and Age-Group (*p* = 0.02, *η*_*p*_^*2*^ = 0.06). Both interactions remained significant when controlling for covariates. **B** There were significant interactions between Time and User-Group (*p* = 0.02, *η*_*p*_^*2*^ = 0.05) and Time and Age-Group (*p* = 0.01, *η*_*p*_^*2*^ = 0.07). Both interactions remained significant when controlling for covariates. **C** There was a significant interaction between Time and Age-Group (*p* = 0.01, *η*_*p*_^*2*^ = 0.06), which remained significant when controlling for covariates. **D** There were no significant effects in the adjusted or unadjusted model. **E** There was a significant interaction between Time and Age-Group (*p* = 0.01, *η*_*p*_^*2*^ = 0.08), which remained significant when controlling for covariates.

Mean activation for each ROI, group, and time-point is displayed in Fig. 1 and Table 2, and full results are presented in supplemental Table 3 (unadjusted) and 4 (adjusted). ICC values and 95% confidence intervals were 0.51 (0.34, 0.64) for right ventral striatum, 0.48 (0.31, 0.62) for left ventral striatum, 0.38 (0.19, 0.54) for right thalamus, 0.38 (0.19, 0.54) for right insula, and 0.50 (0.33, 0.63) for right SMA, reflecting poor-to-moderate reliability.

**Table 2:** Reward anticipation activity in regions of interest by Time, User-Group, and Age-Group.

		Adolescent PWUC (*n* = 26)	Adult PWUC (*n* = 20)	Adolescent controls (*n* = 24)	Adult controls (*n* = 26)	Time x User-Group^a^	Time x Age-Group^a^
		**mean (sd)**	**mean (sd)**	**mean (sd)**	**mean (sd)**	***F (df), p***	***F (df), p***
Right ventral striatum	Baseline	72.98 (44.22)	68.87 (42.28)	64.86 (40.86)	71.92 (34.39)	3.88 (1, 91), 0.05	5.13 (1, 91), 0.03
	Follow-up	70.09 (36.22)	54.32 (42.94)	84.37 (37.20)	66.42 (41.55)		
	Difference	−2.89	−14.55	19.51	−5.50		
Left ventral striatum	Baseline	67.54 (43.27)	68.04 (38.31)	61.93 (41.30)	60.41 (32.14)	5.25 (1, 91), 0.02	6.48 (1, 91), 0.01
	Follow-up	64.33 (33.27)	47.73 (40.38)	78.34 (35.25)	55.26 (32.75)		
	Difference	−3.21	−20.31	16.41	−5.15		
Right thalamus	Baseline	91.17 (65.79)	100.18 (63.23)	80.25 (62.27)	90.77 (43.15)	2.70 (1, 91), 0.10	6.15 (1, 91), 0.01
	Follow-up	91.27 (72.96)	69.85 (58.77)	106.88 (52.35)	79.21 (65.52)		
	Difference	0.10	−30.33	26.63	−11.56		
Right insula	Baseline	68.41 (33.80)	62.36 (38.39)	57.74 (38.80)	57.48 (27.82)	3.62 (1, 91), 0.06	2.74 (1, 91), 0.10
	Follow-up	62.45 (32.18)	48.27 (34.16)	70.92 (29.73)	53.54 (34.44)		
	Difference	−5.96	−14.09	13.18	−3.94		
Right supple-mentary motor area	Baseline	94.38 (48.75)	93.08 (64.24)	82.66 (52.88)	89.09 (45.87)	2.45 (1, 91), 0.12	7.79 (1, 91), 0.01
	Follow-up	102.45 (61.25)	72.21 (54.50)	112.07 (69.63)	82.68 (57.75)		
	Difference	8.07	−20.87	29.41	−6.41		

^a^Adjusted for days/week of cigarette/roll-up use (time-varying), Beck Depression Inventory scores (time-varying), Risk-Taking 18 scores (baseline).

The three-way Time x User-Group x Age-Group interaction was not significant for any region of interest (all *p*s < 0.05).

Both the adjusted and unadjusted models (adjusted estimates presented) showed significant interactions between User-Group and Time for the right (*p* = 0.05, *η*_*p*_^*2*^ = 0.04) and left (*p* = 0.02, *η*_*p*_^*2*^ = 0.05) ventral striatum and significant Time*Age-Group interactions for the right ventral striatum (*p* = 0.03, *η*_*p*_^*2*^ = 0.05), left ventral striatum (*p* = 0.01, *η*_*p*_^*2*^ = 0.07), right thalamus (*p* = 0.01, *η*_*p*_^*2*^ = 0.06), and right SMA (*p* = 0.01, *η*_*p*_^*2*^ = 0.08). There were no significant effects in the right anterior insula (all *p*s > 0.05). As seen in Fig. 1, User-Group effects were driven by decreased activity from baseline to follow-up in PWUC relative to controls, whereas Age-Group effects were driven by increased activity from baseline to follow-up in adolescents relative to adults.

**Fig. 2:**
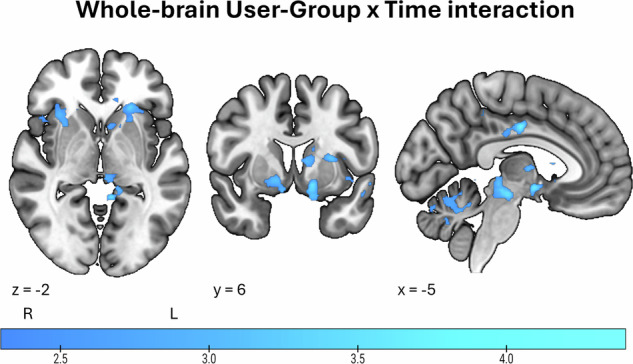
Regions showing reduced reward anticipation activity from baseline to follow-up in PWUC relative to controls in a whole-brain exploratory t-test. PWUC – people who use cannabis*.* Results from a whole-brain independent-samples *t*-test comparing the PWUC and control groups on changes in reward anticipation activity from baseline to follow-up. The analysis was run in the full sample of *n* = 96 and with a cluster-defining threshold *Z* = 2.3.

We additionally performed an exploratory whole-brain independent *t*-test comparing PWUC and controls on changes in activity from baseline to follow-up (using the mid-level ‘difference’ models) at a lower cluster-defining threshold of *Z* = 2.3 [45], to investigate whether our ROI results were replicated in a whole-brain analysis. This analysis showed a decrease in activation from baseline to follow-up among PWUC in five clusters, which partly encompassed our ventral striatum and anterior insula ROIs, as well as the inferior and orbitofrontal cortex, caudate and putamen, midcingulate cortex, and the cerebellum (Fig. 2).

#### Sensitivity and exploratory analyses of change in cannabis use

Fifteen PWUC and 17 controls did not consistently meet the criteria for the PWUC or control group, respectively, over the year. All Time*User-Group and Time*Age-Group interactions in both adjusted and unadjusted ROI analyses remained significant when including only the ‘consistent’ PWUC (17 adolescents, 14 adults) and controls (13 adolescents, 20 adults) (Supplementary Tables 5–7). Additionally, both adjusted and unadjusted analyses showed significant Time*User-Group interactions in the right thalamus (*p* = 0.01, *η*_*p*_^*2*^ = 0.10), right insula (*p* = 0.01, *η*_*p*_^*2*^ *=* 0.10), and right SMA (*p* = 0.03, *η*_*p*_^*2*^* =* 0.07) (adjusted estimates presented).

Mean days/week of cannabis use between baseline and follow-up correlated significantly and negatively with change in activity for all ROIs (Supplementary Fig. 4). Change in days/week of cannabis use between baseline and follow-up did not correlate with change in activity for any ROI, although this variable was influenced by baseline use, as those who used on more days per week at baseline also reduced their frequency of use more (*r* = −0.279, *p* = 0.006). Moreover, although some participants changed their cannabis use throughout the year, days per week of use was strongly correlated across all five sessions (Supplementary Table 8).

## Discussion

In this study, we compared changes in brain reward anticipation activity over one year in adult and adolescent PWUC and controls. ROI analyses showed that activity decreased in the bilateral ventral striatum in the PWUC group relative to the control group. These associations were robust, surviving in both adjusted and unadjusted models and being stronger in sensitivity models including only those who consistently remained a PWUC or control throughout the study period. Adolescents showed an increase and adults a decrease in reward-related brain activity from baseline to follow-up, but there were no significant interactions between cannabis use and age.

Our results suggest that chronic cannabis use is associated with blunted reward anticipation responses in key regions of the brain’s reward system. This was consistent with our hypothesis and with a previous study, which explored the longitudinal association between cannabis use and activity in the brain’s reward system with the MID task [19]. Adolescents showed increased activation from baseline to follow-up, whereas adults exhibited a modest decline, aligning with previous evidence that incentive-related reward system activation strengthens during mid-to-late adolescence [46, 47]. However, we did not find any interactions between user-group and age-group, suggesting that the putative effects of cannabis use and adolescence were additive rather than interactive. In fact, whereas we hypothesised that adolescent PWUC would show a greater decline in reward-related activity than the adult PWUC, our results showed the opposite association; the additive effects of being an adolescent (increase) and using cannabis (decrease) acted in different directions, resulting in the adult PWUC group having the greatest decrease in activity from baseline to follow-up, and the adolescent PWUC showing a similar pattern to adult controls. We did not find evidence that adolescents were more (or less) vulnerable than adults to cannabis-related changes to the reward system, although with *n* = 96 we were likely underpowered to detect a three-way interaction, and future studies in larger samples are needed. Moreover, as younger adolescents may be more vulnerable to cannabis harms than older adolescents, and levels of cannabis use are increasing among older adults in some countries [48, 49], future studies should also explore younger and older age-groups than we included here.

When we only included participants who consistently used cannabis at a rate of ≥1day/week or consistently never used cannabis throughout the study period, we observed significant interactions between time and user-group in the thalamus, insula, and SMA, in addition to the ventral striatum. We also found significant negative correlations between mean days/week of cannabis use between baseline and follow-up and changes in activity in all ROIs in the full sample, suggesting that using cannabis more frequently over the year was associated with a greater reduction in reward anticipation activity across the reward system. These additional findings strengthen our interpretation that the observed reduction in activity was driven by cannabis use. Moreover, our exploratory whole-brain models comparing changes from baseline to follow-up in PWUC and controls found significant (at *Z* = 2.3) group differences in other important reward regions, such as the dorsal striatum and orbitofrontal cortex, and in some non-reward regions [50–52]. Therefore, cannabis use may interact with other components of the reward system in addition to the ventral striatum.

Understanding how the reward system changes with cannabis use can improve interventions for people who want help reducing their use. For instance, although we did not screen participants based on meeting the criteria for cannabis use disorder, our results are consistent with several neurobiological theories [13, 53] and one meta-analysis [15] which suggest that substance and behavioural addiction is characterised by blunted neural responses to non-drug rewards. Blunted reward-related activity in the ventral striatum has also been associated with anhedonia and depression [54, 55]. Effective interventions may therefore need to address the putative reward system imbalance between cannabis and non-cannabis rewards. However, future research is needed to explore whether the reward system in people who use cannabis also responds differently to non-monetary and cannabis rewards, and to determine the psycho-behavioural correlates of blunted reward system responses. Our results can additionally inform brain-based health messaging aimed at reducing the harms of cannabis use.

Major strengths of this study are: (i) the longitudinal design; (ii) the recruitment of PWUC who use cannabis more frequently than in previous studies (3–4 days/week); (iii) careful assessment of cannabis use with the timeline followback; (iv) the novel comparison of adult and adolescent PWUC with age-matched controls; and (v) pre-registration of analyses and hypotheses. Another relevant methodological consideration is that we deliberately did not model the feedback phase of the MID task in the current study. Given that there are twice as many trials that can be analysed for anticipation (all trials, regardless of outcome) than feedback (typically a contrast between successful win and neutral trials or between successful and unsuccessful win trials), and the short interval between the anticipation and feedback phase (2–4 seconds), the task is optimised for detecting brain activity during the anticipation and not the feedback period. This analytical approach, along with the longitudinal design and the fact that we had a larger sample than most previous MID studies in cannabis use, meant that the current study was better powered to detect cannabis-related differences in the reward system compared with previous studies [18].

A limitation of the CannTeen project is the deliberate recruitment of adolescent and adult PWUC, which can produce selection biases. This was necessary to ensure that we had enough people using cannabis frequently, but it also means that our sample is not representative of the general population. Moreover, although we had a large sample compared with previous fMRI studies, it was not sufficient to control for all potential confounders, such as alcohol or illicit drug use. Finally, reliability analyses showed that reward anticipation activity in our five ROIs had only moderate stability from baseline to follow-up. This is consistent with previous research on task-based fMRI [56, 57]

In conclusion, in this 12-month longitudinal fMRI study, PWUC showed reduced reward anticipation activity in the bilateral ventral striatum from baseline to follow-up compared with controls. The effect of cannabis did not interactively differ by age-group, suggesting that 16–17-year-olds were neither more resilient nor more vulnerable to cannabis-related changes in the reward system compared with 26–29-year-olds. However, there was an additive effect such that adult PWUC showed the steepest decline in reward-related brain activity of the four groups. The current results suggest that cannabis use may be prospectively associated with a hypoactive reward system in response to non-drug rewards.

## Supplementary information

Supplementary materials

## Data Availability

Participants in the CannTeen study did not give consent to the sharing of their data outside the research team, and the data are therefore not available.
