# Supplementary material for: The association between cannabis use and brain reward anticipation: a 12-month longitudinal study of adults and adolescents who use cannabis and age-matched controls
Source: Neuropsychopharmacology. 2026 Feb 26;51(9):1538–45. doi: 10.1038/s41386-026-02377-3 (PMC13389008; doi:10.1038/s41386-026-02377-3)
Supplement: Supplementary file 1 — Supplementary materials [file 41386_2026_2377_MOESM1_ESM.docx]

# **SUPPLEMENTAL MATERIALS**

**Title:** The association between cannabis use and brain reward anticipation: a 12-month longitudinal study of adults and adolescents who use cannabis and age-matched controls

**Authors:** Martine Skumlien PhD^1^, Simiao Wang MSc^1^, Tom P Freeman PhD^2^, Molly Eddison MSc^3^, Kat Petrilli PhD^1^, Matthew B Wall PhD^4,5^, Claire Mokrysz PhD^6^, H Valerie Curran PhD^6^, Will Lawn PhD^7^

**Affiliations:**

^1^Department of Addictions, Institute of Psychiatry, Psychology & Neuroscience, King’s College London, London, UK

^2^Addiction and Mental Health Group (AIM), Department of Psychology, University of Bath, Bath, UK

^3^Department of Neuroimaging, Institute of Psychiatry, Psychology & Neuroscience, King’s College London, London, UK

^4^Perceptive, London, UK

^5^Faculty of Medicine, Imperial College London, London, UK

^6^Clinical Psychopharmacology Unit, Clinical, Educational and Health Psychology Department, University College London, London, UK

^7^Department of Psychology, Institute of Psychiatry, Psychology & Neuroscience, King’s College London, London, UK

# **Supplemental Methods**

*The Monetary Incentive Delay task*

Reward anticipation was assessed with the Monetary Incentive Delay (MID) task (1). At the start of each trial, a cue appeared for 500 milliseconds (ms), which signalled whether the participant could win money on that trial (win trials: orange square) or not (neutral trials: blue square). After the cue disappeared there was an anticipation phase for 2-4 seconds (jittered, blank screen), after which a white circle appeared, which the participant responded to by pressing a button on a button-box. Participants were instructed to respond to the white circle as quickly as possible, even if they could not win any money on that trial. After a response had been made, the participants received feedback indicating whether they were successful on that trial, and whether they won any money. Participants could win £0.50 on win trials and their total winnings were calculated and given to them at the end of the session. The required response time for a hit was calibrated to each participant’s performance to obtain a 50% hit rate across all (win and neutral) trials. The initial threshold was set at 300 ms, and was reduced by 16.66 ms (one screen refresh) after each ‘hit’, down to a minimum of 250 ms, or increased by 16.66 ms after each ‘miss’, up to a maximum of 400 ms. There were 66 trials in total, of which 38 were neutral trials and 28 were win trials, with a jittered inter-trial interval between 1.2 and 9.2 seconds. The task took approximately 10 minutes to complete.

*MRI data acquisition and preprocessing*

T_2_* images were acquired using a multiband gradient echo Echo-Planar Imaging (EPI) sequence (repetition time, TR=1250 ms, echo time, TE=30 ms, flip angle=62°, GRAPPA=2) (2). A total of 484 volumes were collected for each participant, with a field-of-view of 192 millimetres (mm) and a matrix size of 64 x 64 mm, and 3 mm isotropic voxels. Forty-four slices were collected using interleaved acquisition, and a multi-band acceleration factor of 2. T_1_-weighted structural images were acquired using a Magnetization Prepared Rapid Gradient Echo (MPRAGE) sequence (3) (TR=2300 ms, TE=2.98 ms, flip angle=9°, parallel imaging acceleration factor=2), with a spatial resolution of 1 mm isotropic voxels.

Structural high-resolution images were pre-processed using the fsl_anat script provided with FSL. Functional images were realigned with MCFLIRT (motion correction FMRIB linear image registration tool) (4) and normalised to MNI-152 (Montreal Neurological Institute) space with FNIRT (FMRIB’s nonlinear registration tool), using a 10 mm warp resolution and 12 degrees of freedom. Spatial smoothing was carried out using a 6 mm full-width at half-maximum Gaussian kernel. Raw functional image series, movement estimates, and registration were carefully inspected for each participant. Seven participants were excluded based on a holistic evaluation of all these factors, as specified in our pre-registered analysis protocol (5).

*Region of interest coordinates*

Regions of interest were selected based on a large meta-analysis of the MID task (6) and defined by constructing 6 mm radii spheres around the coordinates with the peak activation likelihood estimates: right ventral striatum, x = 12, y = 10, z = -4; left ventral striatum, x = -10, y = 10, z = -6; right thalamus, x = 4, y = -18, z = 8; right insula, x = 34, y = 24, z = -2; and the right supplementary motor area, x = 2, y = 6, z = 52.

*Reasons for exclusion*

Participants were excluded for the following reasons, based on our pre-registered protocol (5):

- Excessive movement at baseline, *n* = 4 (one adolescent who used cannabis, two adolescent controls, one adult control);
- Excessive movement at follow-up, *n* = 1 (adult control);
- MRI artefacts at follow-up, *n* = 2 (one adult who used cannabis, one adolescent control)
- Abnormal MID behavioural data at baseline, *n* = 4 (one adolescent who used cannabis, two adults who used cannabis, one adolescent control);
- Abnormal MID behavioural data at follow-up, *n* = 1 (adult control);
- Missing MID behavioural data at follow-up, *n* = 2 (one adolescent who used cannabis, one adult who used cannabis).

Raw functional fMRI image series and movement estimates were inspected for each participant, and the decision to exclude subjects was made based on a holistic evaluation of these factors rather than on a hard cut-off. Participants that were excluded due to movement had maximum movement ≥3 mm in any direction or mean displacement ≥2 mm. Of the five participants excluded due to abnormal behavioural data on the MID task, four had a hit-rate on either win or neutral trials of <25% and one had abnormally low reaction times for win (108 ms) and neutral (114 ms) trials, indicating they had not performed the task correctly.

# **Supplemental Tables**

**Supplemental Table 1.** Full exclusion and exclusion criteria.

|  | Inclusion criteria | Exclusion criteria |
| --- | --- | --- |
| All participants | - Able to come to University College London five times over one year - Capacity to give informed consent - Normal or corrected-to-normal vision - Fluent in English - Right-handed - Able to come to Invicro during normal working hours | - Any illicit drug use within 48 hours of the behavioural baseline session, verified with self-report and saliva testing - Any cannabis or alcohol use within 12 hours of the behavioural baseline session, verified with self-report and saliva/breathalyser testing - Personal history of a diagnosed psychotic episode or disorder - Any one illicit drug taken >2 days/month (averaged over last 3 months) (except laughing gas) - Use of laughing gas >1 day/week (averaged over last 3 months) - Receiving treatment for any mental health condition, including cannabis dependence, in the last month - Unwilling to give blood samples or likely to faint on blood sampling - Current daily use of a medication which is commonly psychotropic - Any mental or physical health problem judged to be problematic for the study, by a medical doctor - Pregnant - Piercings that can’t be removed - Pacemaker - Hearing aid - Full braces - Any other MRI contraindication as judged by Invicro radiographers |
| Adolescent PWUC | - Aged 16-17 years - Cannabis use at a frequency of 1-7 days/week (averaged over last 3 months) | - Age-adjusted BMI <2^nd^ percentile or >99.6^th^ percentile |
| Adolescent controls | - Aged 16-17 years - Between 1 and 10 days of lifetime cannabis use ***or*** 0 days of lifetime cannabis use and at least 1 day of lifetime cigarette/roll-up use (to match groups on the opportunity to use drugs) | - Age-adjusted BMI <2^nd^ percentile or >99.6^th^ percentile - Cannabis use more than once in the last 3 months before behavioural baseline session - Cannabis use in the month prior to the behavioural baseline session |
| Adult PWUC | - Aged 26-29 years - Cannabis use at a frequency of 1-7 days/week (averaged over last 3 months) | - Before the age of 18, cannabis use at a frequency of minimum once per week or more for a period of 3 months or more. - BMI <18.5 or BMI>34.9 |
| Adult controls | - Aged 26-29 years - Between 1 and 10 days of lifetime cannabis use ***or*** 0 days of lifetime cannabis use and at least 1 day of lifetime cigarette/roll-up use (to match groups on the opportunity to use drugs) | - Cannabis use more than once in the last 3 months before behavioural baseline session - Cannabis use in the month prior to the baseline session - BMI <18.5 or BMI>34.9 |

*Abbreviations.* BMI – Body Mass Index, MRI - magnetic resonance imaging, PWUC – people who use cannabis.

**Supplemental Table 2.** Significant results from whole-brain analyses of reward anticipation activity at baseline and follow-up by User-Group and Age-Group.

|  | ***K*** | **x** | **y** | ***z*** | ***Z*** |
| --- | --- | --- | --- | --- | --- |
| **Significant clusters from the paired-samples *t-*test exploring the main effect of Time** | | | | | |
| Frontal pole, right | 678 | 0 | 60 | 10 | 4.3 |
| Frontal pole, right |  | 4 | 64 | 6 | 4.15 |
| Paracingulate gyrus, left |  | -8 | 50 | 8 | 4.1 |
| Frontal pole, right |  | 0 | 58 | 4 | 3.94 |
| Frontal pole, right |  | 6 | 70 | 8 | 3.88 |
| Paracingulate gyrus, right |  | 8 | 50 | 8 | 3.83 |
|  |  |  |  |  |  |
| Frontal pole, right | 676 | 18 | 54 | 42 | 6.16 |
| Frontal pole, right |  | 26 | 36 | 48 | 5.01 |
| Frontal pole, right |  | 20 | 60 | 28 | 4.2 |
| Superior frontal gyrus, right |  | 14 | 36 | 46 | 4.15 |
|  |  |  |  |  |  |
| Frontal pole, left | 577 | -16 | 42 | 48 | 4.77 |
| Superior frontal gyrus, left |  | -16 | 34 | 52 | 4.54 |
| Superior frontal gyrus, left |  | -8 | 36 | 40 | 4.13 |
| Superior frontal gyrus, left |  | -4 | 52 | 42 | 3.98 |
| Middle frontal gyrus, left |  | -34 | 28 | 48 | 3.79 |
| Middle frontal gyrus, left |  | -30 | 30 | 48 | 3.78 |
| **Significant Time*Age-Group clusters from the ANOVA exploring the Time*User-Group, Time*Age-Group, and Time*User-Group*Age-Group interactions** | | | | | |
| Cerebellum, right | 199 | 42 | -72 | -24 | 3.76 |
| Cerebellum, right |  | 16 | -76 | -18 | 3.73 |
| Cerebellum, right |  | 10 | -78 | -20 | 3.66 |
| Occipital fusiform gyrus, right |  | 22 | -74 | -16 | 3.60 |
| Cerebellum, right |  | 48 | -68 | -30 | 3.60 |
| Cerebellum, right |  | 32 | -70 | -20 | 3.45 |
|  |  |  |  |  |  |
| Superior frontal gyrus, left | 177 | -26 | 0 | 74 | 4.49 |
| Superior frontal gyrus, left |  | -20 | 2 | 72 | 4.4 |
| Superior frontal gyrus, left |  | -28 | -4 | 74 | 4.32 |
| Precentral gyrus, left |  | -34 | -8 | 72 | 4.12 |
| Precentral gyrus, left |  | -32 | -10 | 60 | 3.42 |

*Note. K* refers to number of voxels in the cluster and x, y, and z are coordinates in MNI space. Peak *Z*-values are reported for each cluster, and local maxima within clusters.

**Supplemental Table 3.** Unadjusted linear mixed models for reward anticipation activity in regions of interest in the full sample of *n* = 96.

|  | ***F*** | ***df*** | ***p*** | ***η_p_^2^*** |
| --- | --- | --- | --- | --- |
| **Right ventral striatum** |  |  |  |  |
| Time | 0.05 | 1, 92 | .83 | < .01 |
| User-Group | 0.55 | 1, 92 | .46 | .01 |
| Age-Group | 1.15 | 1, 92 | .29 | .01 |
| Time x User-Group | 3.96 | 1, 92 | .05 | .04 |
| Time x Age-Group | 5.39 | 1, 92 | .02 | .06 |
| User-Group x Age-Group | 0.10 | 1, 92 | .76 | < .01 |
| Time x User-Group x Age-Group | 0.71 | 1, 92 | .40 | .01 |
| **Left ventral striatum** |  |  |  |  |
| Time | 0.66 | 1, 92 | .42 | .01 |
| User-Group | 0.10 | 1, 92 | .76 | < .01 |
| Age-Group | 2.36 | 1, 92 | .13 | .03 |
| Time x User-Group | 5.32 | 1, 92 | .02 | .05 |
| Time x Age-Group | 6.57 | 1, 92 | .01 | .07 |
| User-Group x Age-Group | 0.10 | 1, 92 | .75 | < .01 |
| Time x User-Group x Age-Group | 0.09 | 1, 92 | .77 | < .01 |
| **Right thalamus** |  |  |  |  |
| Time | 0.31 | 1, 92 | .58 | < .01 |
| User-Group | 0.01 | 1, 92 | .91 | < .01 |
| Age-Group | 0.49 | 1, 92 | .49 | 0.01 |
| Time x User-Group | 2.75 | 1, 92 | .10 | 0.03 |
| Time x Age-Group | 6.30 | 1, 92 | .01 | 0.06 |
| User-Group x Age-Group | 0.01 | 1, 92 | .91 | < .01 |
| Time x User-Group x Age-Group | 0.08 | 1, 92 | .78 | < .01 |
| **Right insula** |  |  |  |  |
| Time | 0.50 | 1, 92 | .48 | 0.01 |
| User-Group | 0.01 | 1, 92 | .94 | < .01 |
| Age-Group | 2.70 | 1, 92 | .10 | 0.03 |
| Time x User-Group | 3.70 | 1, 92 | .06 | 0.04 |
| Time x Age-Group | 2.75 | 1, 92 | .10 | 0.03 |
| User-Group x Age-Group | 0.01 | 1, 92 | .91 | < .01 |
| Time x User-Group x Age-Group | 0.35 | 1, 92 | .56 | < .01 |
| **Right supplementary motor area** |  |  |  |  |
| Time | 0.20 | 1, 92 | .66 | < .01 |
| User-Group | 0.01 | 1, 92 | .92 | < .01 |
| Age-Group | 1.77 | 1, 92 | .19 | 0.02 |
| Time x User-Group | 2.47 | 1, 92 | .12 | 0.03 |
| Time x Age-Group | 8.07 | 1, 92 | .01 | 0.08 |
| User-Group x Age-Group | 0.04 | 1, 92 | .83 | < .01 |
| Time x User-Group x Age-Group | 0.09 | 1, 92 | .76 | < .01 |

**Supplemental Table 4.** Adjusted linear mixed models for reward anticipation activity in regions of interest in the full sample of *n* = 96.

|  | ***F*** | ***df*** | ***p*** | ***η_p_^2^*** |
| --- | --- | --- | --- | --- |
| **Right ventral striatum** |  |  |  |  |
| Time | 0.04 | 1, 91.73 | .84 | < .01 |
| User-Group | 0.31 | 1, 95.23 | .58 | < .01 |
| Age-Group | 0.89 | 1, 91.39 | .35 | 0.01 |
| BDI | 0.00 | 1, 177.79 | .96 | < .01 |
| RT-18 | 0.11 | 1, 89.81 | .74 | < .01 |
| Cigarette/roll-ups days/week | 0.47 | 1, 166.50 | .49 | < .01 |
| Time x User-Group | 3.88 | 1, 90.61 | .05 | 0.04 |
| Time x Age-Group | 5.13 | 1, 91.19 | .03 | 0.05 |
| User-Group x Age-Group | 0.07 | 1, 89.85 | .79 | < .01 |
| Time x User-Group x Age-Group | 0.70 | 1, 91.98 | .40 | 0.01 |
| **Left ventral striatum** |  |  |  |  |
| Time | 0.59 | 1, 91.77 | .45 | 0.01 |
| User-Group | 0.08 | 1, 95.08 | .78 | < .01 |
| Age-Group | 1.96 | 1, 91.37 | .17 | 0.02 |
| BDI | 0.07 | 1, 179.04 | .79 | < .01 |
| RT-18 | 0.10 | 1, 89.79 | .75 | < .01 |
| Cigarette/roll-ups days/week | 0.09 | 1, 163.97 | .77 | < .01 |
| Time x User-Group | 5.25 | 1, 90.64 | .02 | 0.05 |
| Time x Age-Group | 6.48 | 1, 91.22 | .01 | 0.07 |
| User-Group x Age-Group | 0.14 | 1, 89.83 | .71 | < .01 |
| Time x User-Group x Age-Group | 0.11 | 1, 92.02 | .74 | < .01 |
| **Right thalamus** |  |  |  |  |
| Time | 0.27 | 1, 91.93 | .60 | < .01 |
| User-Group | 0.04 | 1, 94.35 | .85 | < .01 |
| Age-Group | 0.22 | 1, 91.26 | .64 | < .01 |
| BDI | 0.02 | 1, 180.80 | .89 | < .01 |
| RT-18 | 0.65 | 1, 89.68 | .42 | 0.01 |
| Cigarette/roll-ups days/week | 0.13 | 1, 151.50 | .72 | < .01 |
| Time x User-Group | 2.70 | 1, 90.79 | .10 | 0.03 |
| Time x Age-Group | 6.15 | 1, 91.35 | .01 | 0.06 |
| User-Group x Age-Group | 0.07 | 1, 89.71 | .79 | < .01 |
| Time x User-Group x Age-Group | 0.09 | 1, 92.19 | .76 | < .01 |
| **Right insula** |  |  |  |  |
| Time | 0.37 | 1, 91.96 | .55 | < .01 |
| User-Group | 0.02 | 1, 94.18 | .88 | < .01 |
| Age-Group | 2.36 | 1, 91.23 | .13 | 0.03 |
| BDI | 0.49 | 1, 180.27 | .49 | < .01 |
| RT-18 | 0.35 | 1, 89.66 | .55 | < .01 |
| Cigarette/roll-ups days/week | 0.57 | 1, 148.79 | .45 | < .01 |
| Time x User-Group | 3.62 | 1, 90.82 | .06 | 0.04 |
| Time x Age-Group | 2.74 | 1, 91.38 | .10 | 0.03 |
| User-Group x Age-Group | 0.00 | 1, 89.68 | .99 | < .01 |
| Time x User-Group x Age-Group | 0.46 | 1, 92.22 | .50 | < .01 |
| **Right supplementary motor area** |  |  |  |  |
| Time | 0.14 | 1, 91.72 | .71 | < .01 |
| User-Group | 0.07 | 1, 95.25 | .80 | < .01 |
| Age-Group | 0.97 | 1, 91.39 | .33 | 0.01 |
| BDI | 0.37 | 1, 177.58 | .55 | < .01 |
| RT-18 | 0.30 | 1, 89.81 | .59 | < .01 |
| Cigarette/roll-ups days/week | 0.02 | 1, 166.88 | .90 | < .01 |
| Time x User-Group | 2.45 | 1, 90.61 | .12 | 0.03 |
| Time x Age-Group | 7.79 | 1, 91.19 | .01 | 0.08 |
| User-Group x Age-Group | 0.01 | 1, 89.86 | .91 | < .01 |
| Time x User-Group x Age-Group | 0.05 | 1, 91.97 | .83 | < .01 |

*Abbreviations.* BDI – Beck Depression Inventory, RT-18 – Risk-Taking 18.

**Supplemental Table 5.** Participant characteristics for the subsample of *n* = 64 who consistently met the criteria for the PWUC or control group throughout the 12-month study period.

|  | **Adolescent PWUC**  (*n* = 17) | **Adult PWUC**  (*n* = 14) | **Adolescent controls**  (*n* = 13) | **Adult controls**  (*n* = 20) |
| --- | --- | --- | --- | --- |
| **Gender,** n (%) |  |  |  |  |
| Female | 8 (47.1%) | 5 (35.7%) | 4 (30.8%) | 11 (55.0%) |
| Male | 9 (52.9%) | 9 (64.3%) | 9 (69.2%) | 9 (45.0%) |
| **Age in years,** mean (sd) | 17.20 (0.48) | 27.41 (1.01) | 17.25 (0.55) | 27.41 (1.04) |
| **BDI**, mean (sd), range |  |  |  |  |
| Baseline | 7.82 (4.98) | 7.00 (8.57) | 10.54 (5.22) | 6.75 (6.27) |
| Follow-up | 5.71 (4.48) | 9.36 (12.00) | 17.90 (15.90) | 6.45 (8.29) |
| **RT-18**, mean (sd) | 11.24 (2.93) | 7.36 (3.77) | 7.31 (4.57) | 7.90 (4.41) |
| **Cigarette/roll-up use, days/week**, mean (sd), range | | | | |
| Baseline | 3.27 (3.00) | 2.07 (3.06) | 0.97 (2.28) | 0.48 (1.59) |
| Follow-up | 2.67 (2.96) | 1.55 (2.71) | 1.07 (2.61) | 0.64 (1.99) |
| **Number of lifetime cannabis uses**, mean (sd), range |  |  | 3.08 (3.07) | 4.95 (3.15) |
| **Days/week of use**, mean (sd), range | 3.68 (2.05) | 4.52 (1.73) |  |  |
| **CUDIT**, mean (sd), range | 16.00 (6.46) | 12.71 (5.44) |  |  |
| **DSM-5 severe CUD**, n (%) | 7 (41.2%) |  | 3 (21.4%) |  |

*Abbreviations.* BDI – Beck Depression Inventory, CUD – Cannabis Use Disorder, CUDIT – Cannabis Use Disorder Identification Test, DSM – Diagnostic and Statistical Manual of Mental Disorders, PWUC – people who use cannabis, sd – standard deviation, RT-18 – Risk-Taking 18

*Note.* Age was assessed at the baseline imaging session. All other variables were assessed at the baseline behavioural session plus the fifth follow-up behavioural session for the time-varying covariates BDI and days/week of cigarette/roll-up use.

**Supplemental Table 6.** Unadjusted linear mixed models for reward anticipation activity in regions of interest in the subsample of *n* = 64 who consistently met the criteria for the PWUC or control group throughout the 12-month study period.

|  | ***F*** | ***df*** | ***p*** | ***η_p_^2^*** |
| --- | --- | --- | --- | --- |
| **Right ventral striatum** |  |  |  |  |
| Time | 0.33 | 1, 60 | .57 | 0.01 |
| User-Group | 0.25 | 1, 60 | .62 | < .01 |
| Age-Group | 0.68 | 1, 60 | .41 | 0.01 |
| Time x User-Group | 4.48 | 1, 60 | .04 | 0.07 |
| Time x Age-Group | 4.47 | 1, 60 | .04 | 0.07 |
| User-Group x Age-Group | 0.20 | 1, 60 | .65 | < .01 |
| Time x User-Group x Age-Group | 0.29 | 1, 60 | .59 | < .01 |
| **Left ventral striatum** |  |  |  |  |
| Time | 0.71 | 1, 60 | .40 | 0.01 |
| User-Group | < 0.01 | 1, 60 | .97 | < .01 |
| Age-Group | 1.84 | 1, 60 | .18 | 0.03 |
| Time x User-Group | 5.60 | 1, 60 | .02 | 0.09 |
| Time x Age-Group | 6.26 | 1, 60 | .02 | 0.09 |
| User-Group x Age-Group | 0.01 | 1, 60 | .91 | < .01 |
| Time x User-Group x Age-Group | < 0.01 | 1, 60 | .97 | < .01 |
| **Right thalamus** |  |  |  |  |
| Time | 0.30 | 1, 60 | .59 | < .01 |
| User-Group | 0.12 | 1, 60 | .73 | < .01 |
| Age-Group | 0.44 | 1, 60 | .51 | 0.01 |
| Time x User-Group | 6.03 | 1, 60 | .02 | 0.09 |
| Time x Age-Group | 6.70 | 1, 60 | .01 | 0.10 |
| User-Group x Age-Group | < 0.01 | 1, 60 | .96 | < .01 |
| Time x User-Group x Age-Group | 0.10 | 1, 60 | .75 | < .01 |
| **Right insula** |  |  |  |  |
| Time | 0.46 | 1, 60 | .50 | 0.01 |
| User-Group | 2.81 | 1, 60 | .10 | 0.04 |
| Age-Group | 2.32 | 1, 60 | .13 | 0.04 |
| Time x User-Group | 5.64 | 1, 60 | .02 | 0.09 |
| Time x Age-Group | 3.52 | 1, 60 | .07 | 0.06 |
| User-Group x Age-Group | 0.02 | 1, 60 | .89 | < .01 |
| Time x User-Group x Age-Group | 0.08 | 1, 60 | .78 | < .01 |
| **Right supplementary motor area** |  |  |  |  |
| Time | 0.11 | 1, 60 | 0.74 | < .01 |
| User-Group | < 0.01 | 1, 60 | .99 | < .01 |
| Age-Group | 1.22 | 1, 60 | .27 | 0.02 |
| Time x User-Group | 4.78 | 1, 60 | .03 | 0.07 |
| Time x Age-Group | 3.96 | 1, 60 | .05 | 0.06 |
| User-Group x Age-Group | 0.05 | 1, 60 | .83 | < .01 |
| Time x User-Group x Age-Group | 0.02 | 1, 60 | .89 | < .01 |

*Abbreviations.* PWUC – people who use cannabis.

**Supplemental Table 7.** Adjusted linear mixed models for reward anticipation activity in regions of interest in the subsample of *n* = 64 who consistently met the criteria for the PWUC or control group throughout the 12-month study period.

|  | ***F*** | ***df*** | ***p*** | ***η_p_^2^*** |
| --- | --- | --- | --- | --- |
| **Right ventral striatum** |  |  |  |  |
| Time | 0.22 | 1, 60.39 | .64 | < .01 |
| User-Group | 0.25 | 1, 60.65 | .62 | < .01 |
| Age-Group | 0.82 | 1, 58.88 | .37 | 0.01 |
| BDI | 1.07 | 1, 116.52 | .30 | 0.01 |
| RT-18 | 0.70 | 1, 59.33 | .41 | 0.01 |
| Cigarette/roll-ups days/week | 0.99 | 1, 102.26 | .32 | 0.01 |
| Time x User-Group | 5.51 | 1, 60.91 | .02 | 0.08 |
| Time x Age-Group | 4.73 | 1, 58.96 | .03 | 0.07 |
| User-Group x Age-Group | 0.64 | 1, 60.88 | .43 | 0.01 |
| Time x User-Group x Age-Group | 0.63 | 1, 62.35 | .43 | 0.01 |
| **Left ventral striatum** |  |  |  |  |
| Time | 0.71 | 1, 60.44 | .40 | 0.01 |
| User-Group | < 0.01 | 1, 60.47 | .96 | < .01 |
| Age-Group | 1.78 | 1, 58.82 | .19 | 0.03 |
| BDI | 0.02 | 1, 117.00 | .87 | < .01 |
| RT-18 | 0.10 | 1, 59.25 | .75 | < .01 |
| Cigarette/roll-ups days/week | 0.38 | 1, 98.55 | .54 | < .01 |
| Time x User-Group | 5.76 | 1, 60.96 | .02 | 0.09 |
| Time x Age-Group | 6.17 | 1, 59.03 | .02 | 0.09 |
| User-Group x Age-Group | 0.03 | 1, 60.83 | .85 | < .01 |
| Time x User-Group x Age-Group | < 0.01 | 1, 62.45 | 1.00 | < .01 |
| **Right thalamus** |  |  |  |  |
| Time | 0.24 | 1, 60.53 | .63 | < .01 |
| User-Group | 0.02 | 1, 59.91 | .89 | < .01 |
| Age-Group | 0.30 | 1, 58.60 | .59 | 0.01 |
| BDI | 0.43 | 1, 113.15 | .51 | < .01 |
| RT-18 | 2.44 | 1, 58.99 | .12 | 0.04 |
| Cigarette/roll-ups days/week | 0.67 | 1, 88.01 | .41 | 0.01 |
| Time x User-Group | 6.51 | 1, 61.01 | .01 | 0.10 |
| Time x Age-Group | 6.66 | 1, 59.22 | .01 | 0.10 |
| User-Group x Age-Group | 0.32 | 1, 60.56 | .57 | 0.01 |
| Time x User-Group x Age-Group | 0.21 | 1, 62.60 | .65 | < .01 |
| **Right insula** |  |  |  |  |
| Time | 0.33 | 1, 60.54 | .57 | 0.01 |
| User-Group | 2.08 | 1, 59.82 | .15 | 0.03 |
| Age-Group | 2.39 | 1, 58.56 | .13 | 0.04 |
| BDI | 1.24 | 1, 111.97 | .27 | 0.01 |
| RT-18 | 1.73 | 1, 58.95 | .19 | 0.03 |
| Cigarette/roll-ups days/week | 1.13 | 1, 86.54 | .29 | 0.01 |
| Time x User-Group | 6.62 | 1, 61.01 | .01 | 0.10 |
| Time x Age-Group | 3.70 | 1, 59.25 | .06 | 0.06 |
| User-Group x Age-Group | 0.45 | 1, 60.51 | .50 | 0.01 |
| Time x User-Group x Age-Group | 0.28 | 1, 62.60 | .60 | < .01 |
| **Right supplementary motor area** |  |  |  |  |
| Time | 0.09 | 1, 60.41 | .76 | < .01 |
| User-Group | 0.05 | 1, 60.57 | .83 | < .01 |
| Age-Group | 0.81 | 1, 58.85 | .37 | 0.01 |
| BDI | 0.09 | 1, 116.85 | .77 | < .01 |
| RT-18 | 1.60 | 1, 59.29 | .21 | 0.03 |
| Cigarette/roll-ups days/week | 0.14 | 1, 100.60 | .71 | < .01 |
| Time x User-Group | 4.87 | 1, 60.93 | .03 | 0.07 |
| Time x Age-Group | 3.91 | 1, 58.99 | .05 | 0.06 |
| User-Group x Age-Group | 0.35 | 1, 60.86 | .56 | 0.01 |
| Time x User-Group x Age-Group | < 0.01 | 1, 62.40 | .95 | < .01 |

*Abbreviations.* BDI – Beck Depression Inventory, PWUC – people who use cannabis, RT-18 – Risk-Taking 18.

**Supplemental Table 8.** Pearson correlations between days per week of cannabis use at each session.

|  | Mean days/week session 2-5 | Days/week change session 2-5 vs. 1 | Days/week session 1 | Days/week session 2 | Days/week session 3 | Days/week session 4 |
| --- | --- | --- | --- | --- | --- | --- |
| Days/week change session 2-5 vs. 1 | *r* = .200  *p* = .05 |  |  |  |  |  |
| Days/week session 1 | *r* = .885  *p* < .001 | *r* = -.279  *p* = .006 |  |  |  |  |
| Days/week session 2 | *r* = .957  *p* < .001 | *r* = .012  *p* = .91 | *r* = .932  *p* < .001 |  |  |  |
| Days/week session 3 | *r* = .973  *p* < .001 | *r* = .121  *p* = .27 | *r* = .879  *p* < .001 | *r* = .943  *p* < .001 |  |  |
| Days/week session 4 | *r* = .967  *p* < .001 | *r* = .303  *p* = .005 | *r* = .820  *p* < .001 | *r* = .876  *p* < .001 | *r* = .915  *p* < .001 |  |
| Days/week session 5 | *r* = .943  *p* < .001 | *r* = .279  *p* = .007 | *r* = .779  *p* < .001 | *r* = .837  *p* < .001 | *r* = .860  *p* < .001 | *r* = .928  *p* < .001 |

# **Supplemental Figures**

**
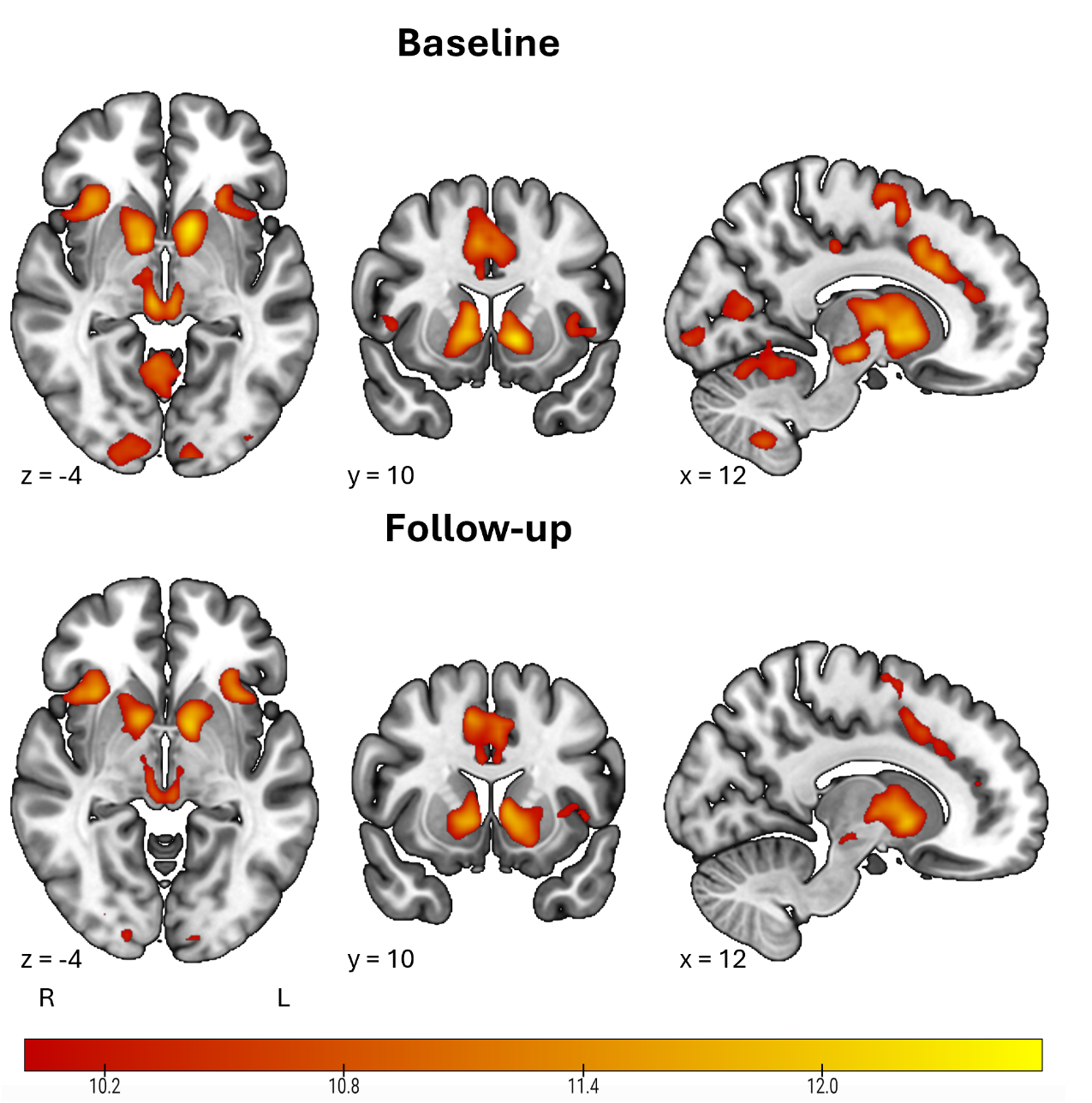
**

**Supplemental Figure 1. Reward anticipation activity in the full sample at baseline and follow-up.**

*Note*. Results from a one-sample *t*-test of reward anticipation in the full sample of *n* = 96 participants. Images are presented in radiological orientation, such that left on the image is the right hemisphere. A threshold of *Z* = 10 was used for visualisation purposes.


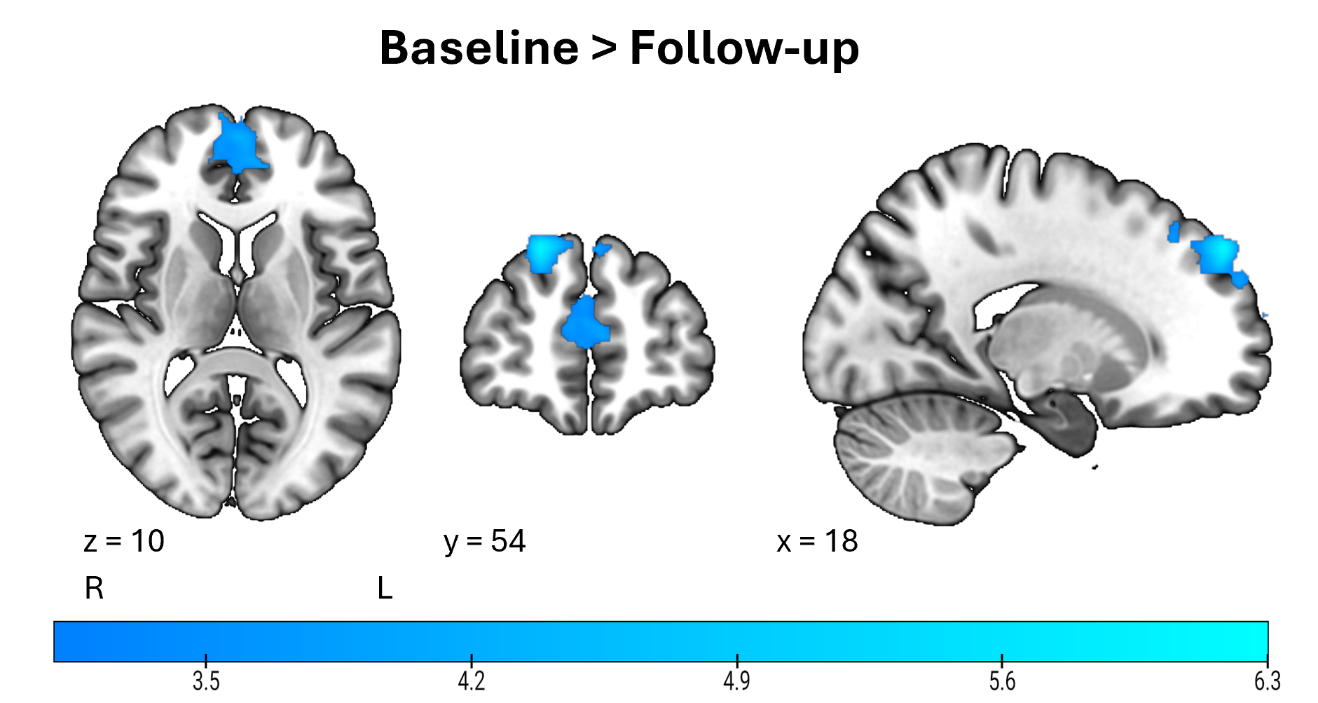


**Supplemental Figure 2. Differences in reward anticipation activity at baseline and follow-up in the full sample.**

*Note*. Results from a whole-brain paired-samples *t*-test comparing baseline and follow-up activity in the full sample of *n* = 96 (cluster-defining threshold *Z* = 3.1). Images are presented in radiological orientation, such that left on the image is the right hemisphere.

**
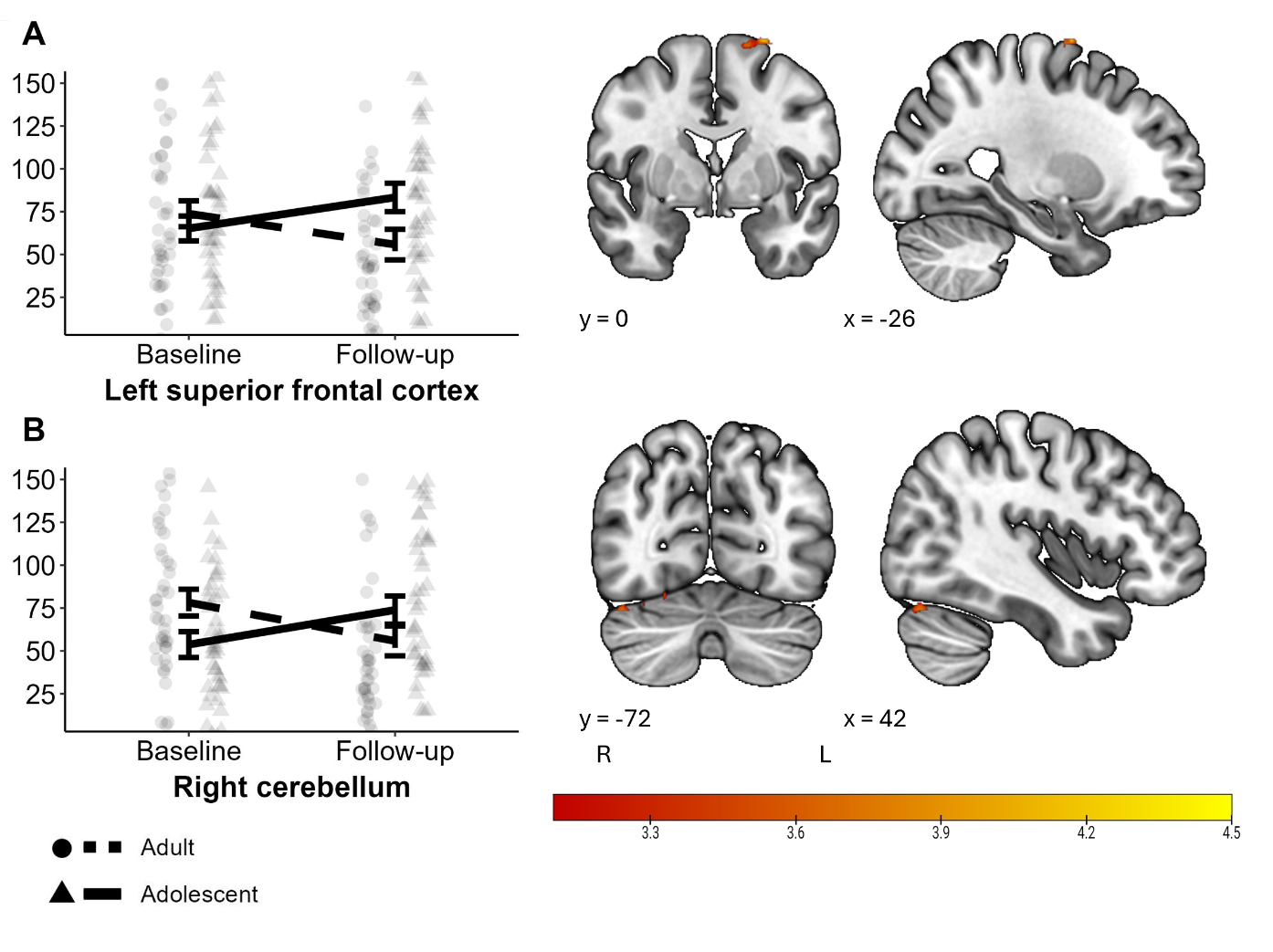
**

**Supplemental Figure 3. Differences in reward anticipation activity at baseline and follow-up by Age-Group.**

*Note*. Significant clusters from a whole-brain 2X2 analysis of variance with User-Group, Age-Group, and User-Group*Age-Group, using mid-level models comparing baseline and follow-up activity in the full sample of *n* = 96 (cluster-defining threshold *Z* = 3.1). Images are presented in radiological orientation, such that left on the image is the right hemisphere. **A.** Significant Time*Age-Group interaction in the left superior frontal cortex. **B.** Significant Time*Age-Group interaction in the right cerebellum.

*
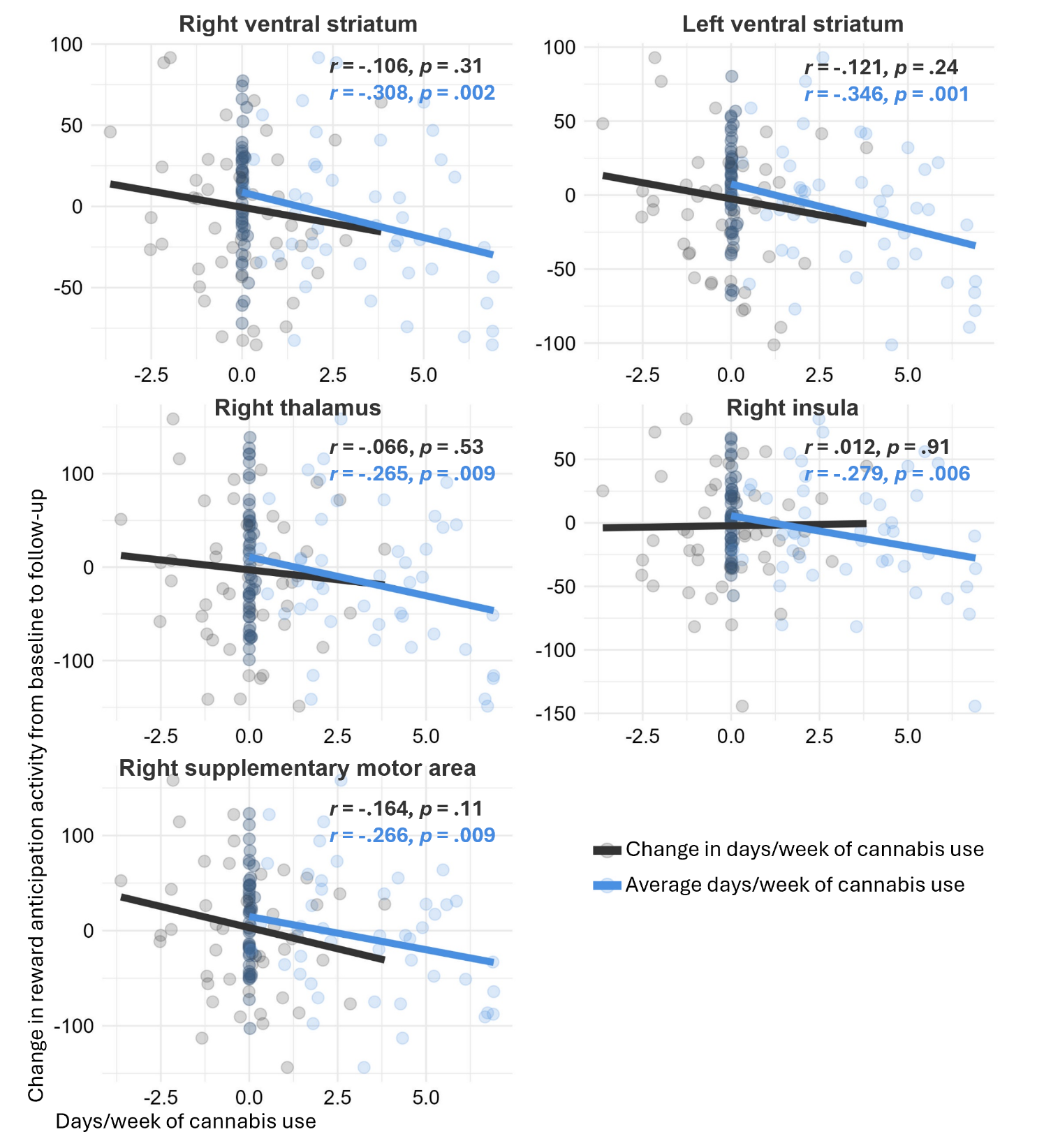
*

**Supplemental Figure 4. Bivariate Pearson correlations between change scores for each region of interest and change in and average days per week of cannabis use between baseline and follow-up.**

*Note*. Change scores for regions of interest were computed by subtracting activation at baseline from activation at follow-up. Average days/week of cannabis use was computed by taking the mean of all behavioural follow-up sessions (sessions 2-5) that the participant attended. Change in days/week of cannabis use was computed by subtracting baseline (session 1) days/week of use from this average.

# **References**

1. Knutson B, Westdorp A, Kaiser E, Hommer D. FMRI visualization of brain activity during a monetary incentive delay task. Neuroimage. 2000;12(1):20-7. doi:10.1006/nimg.2000.0593

2. Demetriou L, Kowalczyk OS, Tyson G, Bello T, Newbould RD, Wall MB. A comprehensive evaluation of increasing temporal resolution with multiband-accelerated protocols and effects on statistical outcome measures in fMRI. Neuroimage. 2018;176:404-16. doi:10.1016/j.neuroimage.2018.05.011

3. Jack CR, Jr., Bernstein MA, Fox NC, Thompson P, Alexander G, Harvey D, et al. The Alzheimer's Disease Neuroimaging Initiative (ADNI): MRI methods. J Magn Reson Imaging. 2008;27(4):685-91. doi:10.1002/jmri.21049

4. Jenkinson M, Bannister P, Brady M, Smith S. Improved optimization for the robust and accurate linear registration and motion correction of brain images. Neuroimage. 2002;17(2):825-41. doi:10.1016/s1053-8119(02)91132-8

5. Wang S, Lawn W, Skumlien M, Wall MB, Freeman TP, Curran HV. How do the neural correlates of reward anticipation change over one year in adolescent and adult cannabis users and age-matched controls? 2022. <https://osf.io/updvg/>.

6. Oldham S, Murawski C, Fornito A, Youssef G, Yücel M, Lorenzetti V. The anticipation and outcome phases of reward and loss processing: A neuroimaging meta-analysis of the monetary incentive delay task. Hum Brain Mapp. 2018;39(8):3398-418. doi:10.1002/hbm.24184
